# The neurophysiology of intraoperative error: An EEG study of trainee surgeons during robotic-assisted surgery simulations

**DOI:** 10.3389/fnrgo.2022.1052411

**Published:** 2023-01-09

**Authors:** Christopher D'Ambrosia, Eliah Aronoff-Spencer, Estella Y. Huang, Nicole H. Goldhaber, Henrik I. Christensen, Ryan C. Broderick, Lawrence G. Appelbaum

**Affiliations:** ^1^College of Physicians and Surgeons, Columbia University, New York, NY, United States; ^2^Cognitive Robotics Laboratory, Department of Computer Science and Engineering, Contextual Robotics Institute, University of California, San Diego, La Jolla, CA, United States; ^3^Department of Medicine, University of California, San Diego, La Jolla, CA, United States; ^4^Division of Minimally Invasive Surgery, Department of Surgery, University of California, San Diego, La Jolla, CA, United States; ^5^Department of Psychiatry, University of California, San Diego, La Jolla, CA, United States

**Keywords:** robot-assisted surgery, medical robotics, human performance, surgical training, clinical assessment

## Abstract

Surgeons operate in mentally and physically demanding workspaces where the impact of error is highly consequential. Accurately characterizing the neurophysiology of surgeons during intraoperative error will help guide more accurate performance assessment and precision training for surgeons and other teleoperators. To better understand the neurophysiology of intraoperative error, we build and deploy a system for intraoperative error detection and electroencephalography (EEG) signal synchronization during robot-assisted surgery (RAS). We then examine the association between EEG data and detected errors. Our results suggest that there are significant EEG changes during intraoperative error that are detectable irrespective of surgical experience level.

## 1. Introduction

Surgeons operate in mentally and physically demanding workspaces where the impact of error is highly consequential (Christian et al., 2006; Modi et al., 2018). The cognitive and affective states of surgeons in the operating room (OR) are primary determinants of surgical performance (Schuetz et al., 2008; Yurko et al., 2010; Haji et al., 2015). Measuring changes in these states contributes to insightful performance analysis, training development and skills improvement (Eversbusch and Grantcharov, 2004; Carswell et al., 2005). However, these cognitive and affective state measurements, which can be derived from neurophysiological metrics, must be coupled with measures of surgical task success or failure to enable any conclusions about the correspondences between psychological indicators and surgical performance (Modi et al., 2017). Unfortunately, the majority of existing tools to measure surgical task performance are retrospective, reliant on subjective, manual expert annotation and often focused on entire procedures rather than on intraoperative performance (Maruthappu et al., 2014).

Examining the associations between neurophysiology and intraoperative performance requires detecting markers of intraoperative success or failure at millisecond to second resolutions and synchronizing those markers with neurophysiological data. In this study, we build and deploy an automated, objective error detection system for robotic-assisted surgery (RAS) simulations that is synchronized with an electroencephalogram (EEG) headset worn by trainee surgeons at an RAS operating console. We use this system to detect intraoperative errors and analyze the accompanying EEG changes during those errors.

Prior work examining EEG changes during RAS has primarily focused on correlating EEG measurements with retrospective, subjective surveys of perceived mental workload (Guru et al., 2015b; Yu et al., 2017; Zander et al., 2017; Zhou et al., 2018, 2020). While mental workload is a significant contributor to surgical performance (Sarker and Vincent, 2005; Healey et al., 2006; Balch et al., 2009), it is not a proxy for actual performance. Understanding the relationship between operator neurophysiology and actual surgical performance requires objective, high temporal-resolution measurements of intraoperative performance that can be synchronized with changes in neurophysiological indicators. Automated skill and error assessment algorithms for RAS platforms do exist (Chen et al., 2019) but are rarely used in conjunction with biometric data capture. Integrating intraoperative performance data with intraoperative physiological data enables investigation of the potential cognitive and affective features that influence surgical success or failure.

For neurophysiological assessment, we choose to focus on the EEG indicators of psychological features that have been shown to impact task performance in mentally and physically demanding workspaces as well as human-machine teams. “Focused attention” or “vigilance” is the ability of an individual to maintain sustained attention on a task (Oken et al., 2006). Decreases in focused attention increase error rates in human-machine teamwork (Gill, 2012). Fluctuations in levels of attention can be measured through changes in EEG spectral power analyses (Borghini et al., 2014). For this study, we examine global β: (α+θ) power ratio (Pope et al., 1995), occipital β power (Gola et al., 2013), occipital δ power (Harmony, 2013), occipital θ power (Putman et al., 2014), frontal θ power (Strijkstra et al., 2003), and global γ power (Kaiser and Lutzenberger, 2005) as quantitative indicators of attention.

Cognitive load and fatigue are similarly relevant to human-robot teaming. High levels of cognitive load lead to task errors (Sweller, 2011). Cognitive fatigue, a decrease in cognitive performance after sustained cognitive load (Ackerman, 2011), is of particular importance in safety-critical aviation, transportation, aerospace, military, medicine, and industrial settings where fatigued individuals routinely operate complex, automated systems (Zhang et al., 2009). EEG spectral power ratios (Ryu and Myung, 2005) can also be used as measures of cognitive load or fatigue. We focus on frontal θ power (Shou and Ding, 2013; So et al., 2017; Raufi and Longo, 2022), frontal α power (Palva and Palva, 2007) and parietal α power (Frey et al., 2015) as indicators of cognitive load. For cognitive fatigue, we examine global α power (Santamaria and Chiappa, 1987), occipital α power (Thut et al., 2006; Zumer et al., 2014), frontal θ power (Strijkstra et al., 2003), and θ:α power ratio at Fz (Berka et al., 2007).

Affective characteristics such as valence, arousal, and dominance (VAD) may also influence human-robot system performance. Valence, the positivity or negativity of emotion, modulates attention and has shown an inverted u-shaped relationship with human performance in various tasks (Cai and Lin, 2011). Arousal, often referred to as “stress,” has a similar inverted u-shaped relationship with cognitive load (Teigen, 1994; Staal, 2004). Dominance, feelings of being “in control” of a situation, has a positive relationship with performance during sustained tasks (Cohen et al., 2016). EEG correlates of valence include α power asymmetry (Ohme et al., 2010; Gordon et al., 2018), global β power (Liu and Sourina, 2013), global γ power (Oathes et al., 2008) and global α power (Ahern and Schwartz, 1985). Indicators of arousal include global θ power, global β power and global γ power (Liu and Sourina, 2013). EEG correlates of dominance include global α:β power ratio and parietal β power (Verma and Tiwary, 2017).

Finally, we examine the association of proposed error recognition-related EEG metrics with intraoperative error. We include global θ power (Cavanagh and Frank, 2014), and global β power (Ray and Cole, 1985) as quantitative EEG indicators of error recognition.

To correlate these EEG metrics with markers of intraoperative error, we require high temporal-resolution analysis of surgical performance data. Prior work in automated surgical performance analysis has primarily focused on kinematic, system event, and haptic data obtained from RAS platforms. This data is then used to find associations between features such as instrument traveling distance (kinematics), camera clutch engagement (system event), and grip force (haptics) and self-reported level of surgical experience (Chen et al., 2019). Other research has used surgical video analysis for competency assessment (Reiley and Hager, 2009; Tao et al., 2012; Wang and Majewicz Fey, 2018). While kinematic, system, haptic, and image data can be correlated with surgical experience and used for skills assessments, in order to examine the EEG correlates of actual task errors, automated surgical error detection is required. Prior work in intraoperative error detection largely relies on retrospective, manual annotations by expert surgeons (Chen et al., 2019). These manual annotations provide sub-task level resolution for error detection but sub-tasks within surgical procedures can take seconds to minutes. A sub-task that is labeled with an error then implies an error marker of seconds to minutes in length. The EEG signal associated with a long-duration error marker would include more than the neurophysiological response to error.

To more precisely examine the neurophysiology of intraoperative error, we use a video analysis pipeline to automatically label each video frame from all surgical simulation videos for the presence or absence of an error. Because RAS operating console video is captured at a frame rate of 30 frames-per-second (fps) and each frame has an error label, we achieve error detection resolution of 30 Hertz (Hz). Each of these error labels is associated with a timestamp that is then synchronized with raw EEG data. By creating non-overlapping EEG data windows that align with each video frame, we create a more accurate measure of the neurophysiology of intraoperative error.

Parameterizing the EEG-based neurophysiology of intraoperative error creates the possibility of designing training interventions to reduce or prevent these errors. Surgeons who exhibit high correlations between EEG indices that may be related to attention and error, cognitive load and error, or emotional valence and error, for example, may benefit from EEG neurofeedback training to improve attention (Egner and Gruzelier, 2001, 2004), cognitive load management (Gruzelier et al., 2006), memory (Vernon et al., 2003), or emotional regulation (Raymond et al., 2005). Other surgeons who have high correlations between EEG indicators of arousal or stress and error, or sensorimotor desynchronization and error, for example, may benefit from focused neurostimulation for stress reduction (Modi et al., 2019) and fine motor skill augmentation (Cox et al., 2020).

In this paper, we aim to characterize the neurophysiological response to intraoperative error during RAS. To achieve this, we build and deploy a system for intraoperative error detection and EEG signal synchronization. We then use this system in a series of experiments with trainee surgeons and analyze the results. Our motivation is a better understanding of the neurophysiology of intraoperative error to help guide more accurate human performance assessments and training for surgeons and other teleoperators.

## 2. Materials and methods

### 2.1. Materials

We use a daVinci Xi operating console (Intuitive Surgical, Inc.) for image-based surgical data. The Xi operating console provides video of the surgeon's field-of-view (FOV) at 30 fps. Video analysis of the operating console video (surgeon's FOV) is used for intraoperative error detection. For neurophysiological data capture, we use a Cognionics Quick-20 EEG headset (Cognionics, Inc.) with a sampling rate of 500 Hz.

### 2.2. Methods

#### 2.2.1. Experimental protocol

Twenty participants were recruited for this study under institutional IRB approval. Of these participants, 5 were non-medical graduate students and the remainder were general surgery residents at various levels of training. All participants were naïve to the hypotheses of the study prior to participation.

**Figure 1:**
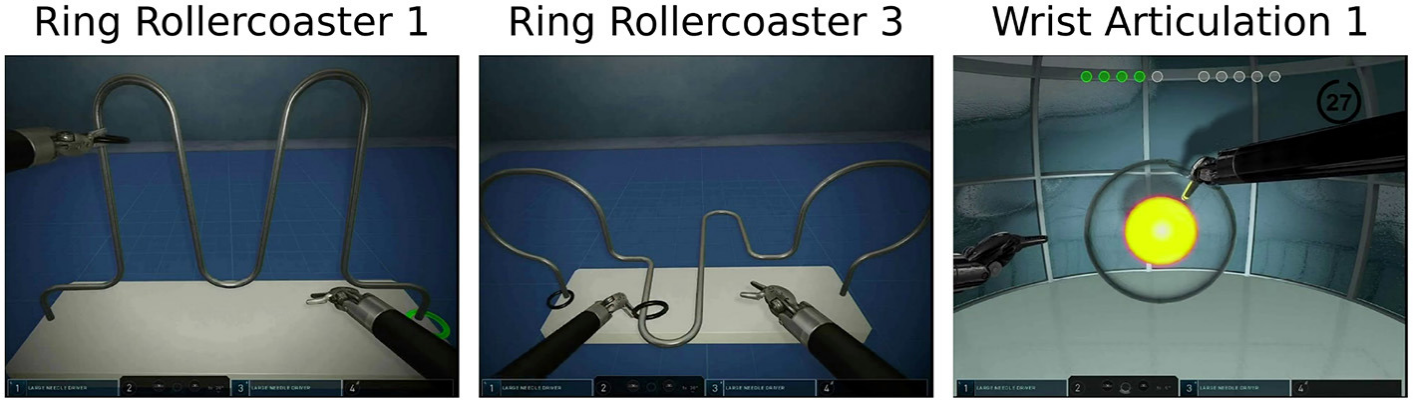
Surgical simulation tasks.

Each participant completed three simulation tasks during a single sitting without breaks. These tasks are standard exercises used in robotic surgical training to teach surgeons operational procedures for robot-assisted surgery. They are part of a larger standard learning curriculum that is widely used in surgical education curricula that are designed, tested, and deployed to improve robot assisted surgery skills. These tasks were completed in the same order for all participants. The tasks utilized were “Ring Rollercoaster 1,” “Ring Rollercoaster 3,” and “Wrist Articulation 1” in this order for all participants (Figure 1).

**Figure 2:**
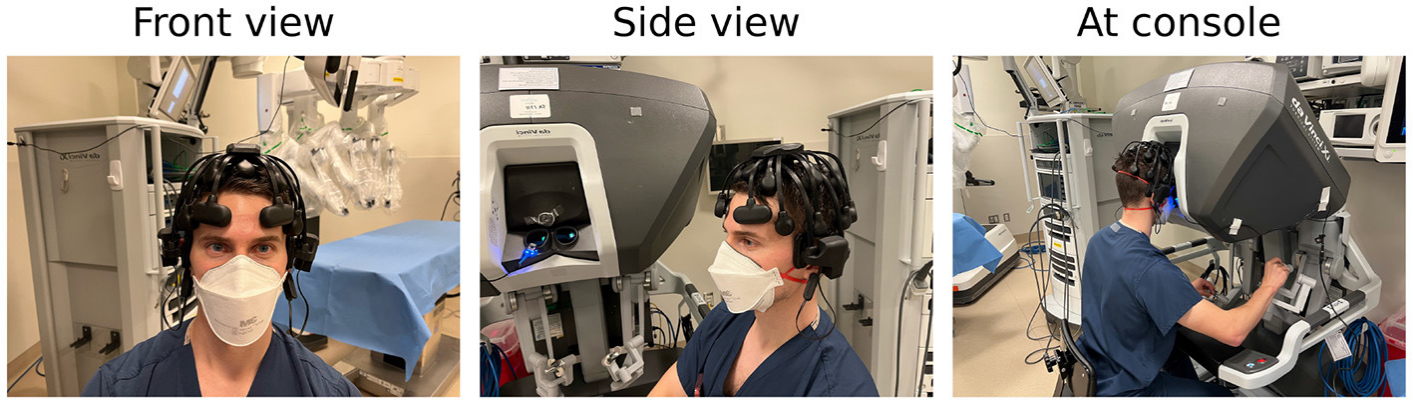
EEG headset deployed in RAS-equipped OR.

An EEG headset was fitted on each participant prior to beginning the first task and checked for appropriate positioning and data transfer. We use the international 10–20 EEG system to ensure proper electrode placement. An example of a deployed headset is shown in Figure 2. Following appropriate electrode placement, we confirm adequate signal through the Cognionics Data Aquisition software (Cognionics, Inc.) which provides visual cues to monitor signal quality at individual EEG electrodes. Electrodes with low signal quality are re-adjusted until signal quality meets the data acquisition software criteria. This signal quality is monitored throughout the experiment, and the EEG headset is re-adjusted between simulation tasks if necessary.

Four minutes of baseline EEG readings were then recorded as each participant sat still at the operating console. Following baseline recording, each participant was directed to start the first surgical simulation task and given no other instruction. After completion of the first task, each participant was directed to start the second simulation, also without additional instruction or feedback. After completion of the second simulation, each participant was directed to start the third and final simulation, also without additional instruction or feedback. Following completion of the final simulation, each participant was instructed to complete a demographics survey after which the EEG headset was removed and the system was reset for future participants.

#### 2.2.2. Video analysis

To generate error markers from operating console (i.e., surgeon FOV) video, we use each video frame as input for a classical computer vision feature extraction algorithm. Our algorithm uses pixel-based frequency filters, intensity filters and Hough transforms to generate a feature representation for each video frame. This feature representation is used to label each video frame for the presence or absence of intraoperative error. The types of error we consider are: contact between the manipulated object and an obstacle, excessive grip force, dropping the manipulated object, contact between end effectors, end effectors going out of view during camera clutch movement, contact between end effectors and obstacles.

To validate this video analysis pipeline, a single expert human adjudicator manually annotated a validation set of 18,887 randomly chosen operating console video frames for the presence of intraoperative error. We compared these manual annotations to the annotations generated by our computer vision algorithm on the same validation set of operating console video frames. Our algorithm's overall error detection accuracy on the validation set of 18,887 operating console video frames is 98.0%, precision is 90.4%, and recall is 93.7%. We then use this pipeline for frame-by-frame inference on the video data. Given the operating console video frame rate of 30 fps, frame-by-frame error detection provides intraoperative error markers at 30 Hz.

### 2.3. EEG analysis

To pre-process the raw EEG data, we remove the DC offset and bandpass filter the resulting signal with a passband of 0.01–50 Hz. We eliminate EEG noise using artifact subspace reconstruction (Chang et al., 2018). This pre-processed data is then filtered in parallel into five spectral power bands: delta (1–4 Hz), theta (4–8 Hz), alpha (8–12 Hz), beta (12–30 Hz), and gamma (30–50 Hz). This filtering is done on the entire EEG data stream i.e., from the start of the baseline or start of the simulation period to the end of the baseline or end of the simulation period. After this filtering is complete, the EEG data for each of the spectral power bands is then windowed.

To accurately associate each operating console video frame with a non-overlapping window of EEG data, we align each video frame timestamp with the closest EEG signal timestamp. This timestamp is the “start window” timestamp. Each EEG window includes all samples received between a “start window” timestamp and the “start window” timestamp corresponding to the next video frame. The operating console video has a frame rate of 30 fps which implies that each video frame has a duration of ~33 ms. Each EEG window therefore has a size of ~33 ms.

Using these non-overlapping ~33 ms EEG windows, we compute the root-mean-square amplitude of each band for every EEG channel. Channel-specific spectral band powers for each window (i.e., video frame) are then aggregated into features that correspond to each operating console video frame. These features are compared to the average feature value for the baseline period.

### 2.4. Statistical analysis

For analysis of all EEG channels and all spectral power bands, we use separate linear mixed effects models for each channel and each band. Given 19 EEG channels and 5 spectral power bands, this results in 95 mixed effects models. In each of these 95 models, the dependent variable (channel-band power) is standardized, intraoperative error is modeled as a fixed effect and individual participant variance is modeled as a random effect.

For EEG feature analysis, we examine a total of 15 distinct EEG features. We select these 15 features (see Table 1 for all features) based on previously published work from other contexts that associates these EEG features with cognitive and affective attributes that could be important during RAS. We do not attempt to reproduce earlier work from other contexts by testing whether these EEG features are appropriate indicators of cognition or affect in an RAS simulation. Instead, we test whether these 15 EEG features are associated with intraoperative error. We use a separate linear mixed effects model for each EEG feature. The dependent variable (EEG feature) is standardized, intraoperative error is modeled as a fixed effect and individual participant variance is modeled as a random effect.

**Table 1:** EEG correlates of selected cognitive and affective features from previously published research.

**Cognitive/affective feature**	**EEG correlate**	**Spectral bands: EEG electrodes**
Attention	Global β: (α+θ) power ratio (Pope et al., 1995)	β, α, θ: All
	Occipital β power (Gola et al., 2013)	β: O1, O2
	Occipital δ power (Harmony, 2013)	δ: O1, O2
	Occipital θ power (Putman et al., 2014)	θ: O1, O2
	Frontal θ power (Strijkstra et al., 2003)	θ: F4, Fz, F3
	Global γ power (Kaiser and Lutzenberger, 2005)	γ: all
Cognitive load	Frontal θ power (Shou and Ding, 2013; So et al., 2017; Raufi and Longo, 2022)	θ: F4, Fz, F3
	Frontal α power (Palva and Palva, 2007)	α: F4, Fz, F3
	Parietal α power (Frey et al., 2015)	α: P3, P4, Pz, P7, P8
Cognitive fatigue	Global α power (Santamaria and Chiappa, 1987)	α: All
	Occipital α power (Thut et al., 2006; Zumer et al., 2014)	α: O1, O2
	Frontal θ power (Strijkstra et al., 2003)	θ: F4, Fz, F3
	θ:α power ratio (Berka et al., 2007)	θ, α: Fz
Valence	α power asymmetry (Ohme et al., 2010; Gordon et al., 2018)	α: (F7, Fp1, F3, C3, P7, T3, P3, O1) / (Fp2, F8, F4, P8, P4, O2, C4, T4)
	Global β power (Liu and Sourina, 2013)	β: all
	Global γ power (Oathes et al., 2008)	γ: all
	Global α power (Ahern and Schwartz, 1985)	α: all
Arousal	Global θ power	θ: all
	Global β power	β: all
	Global γ power (Liu and Sourina, 2013)	γ: all
Dominance	Global α:β power ratio	α, β: all
	Parietal β power (Verma and Tiwary, 2017)	β: P3, P4, Pz, P7, P8
Error recognition	Global θ power (Cavanagh and Frank, 2014)	θ: all
	Global β power (Ray and Cole, 1985)	β: all

All electrodes: Fp1, Fp2, F7, F3, Fz, F4, F8, T3, C3, Cz, C4, T4, P7, P3, Pz, P4, P8, O1, and O2.

Given the independent testing of 95 channel-band combinations as well as 15 EEG features in this experiment, we correct our baseline significance level of *P* = 0.01 by a factor of $\frac{1}{110}$. Our multiple comparisons corrected significance level is *P* = 0.00009.

We conducted a simulation-based power analysis (Green and MacLeod, 2016) of the EEG feature correlates using simulated effect sizes approximately equal to those reported in our results (Brysbaert and Stevens, 2018) and significance levels of *P* = 0.00009. With a simulated effect size of 0.05, our statistical power to detect error-related changes in occipital delta band power has a 95% confidence interval (C.I.) of 99.6–100.0%. With a simulated effect size of –0.03, the 95% statistical power C.I. for parietal alpha band power is 99.6 to 100.0%. With a simulated effect size of 0.03, the 95% statistical power C.I. for frontal theta band power is 99.6–100.0%. With a simulated effect size of –0.05, the 95% statistical power C.I. for left-to-right alpha band power asymmetry is 99.6–100.0%. With a simulated effect size of –0.02, the 95% statistical power C.I. for global beta band power is 99.3–99.9%. With a simulated effect size of –0.04, the 95% statistical power C.I. for parietal beta band power is 99.6–100.0%.

## 3. Results

### 3.1. Video analysis

**Figure 3:**
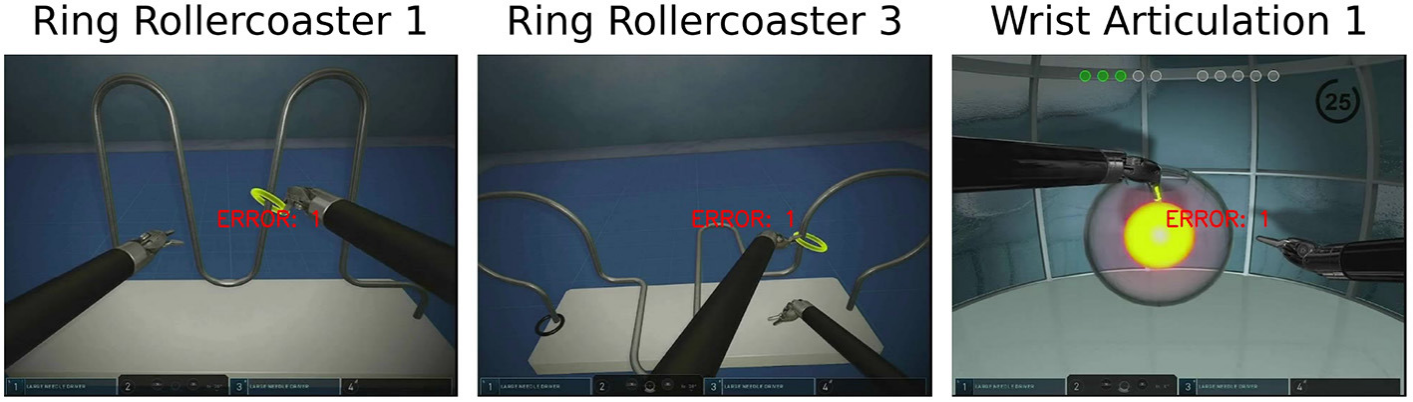
Error detection example for simulation tasks.

The sample size of detected errors using was smaller than that of the non-errors. There were a total of 62,809 detected error frames (17.1%) as compared to 305,234 non-error frames (82.9%). See Figure 3 for example error detection frames from each simulation task.

### 3.2. All channels all bands analysis

**Figure 4:**
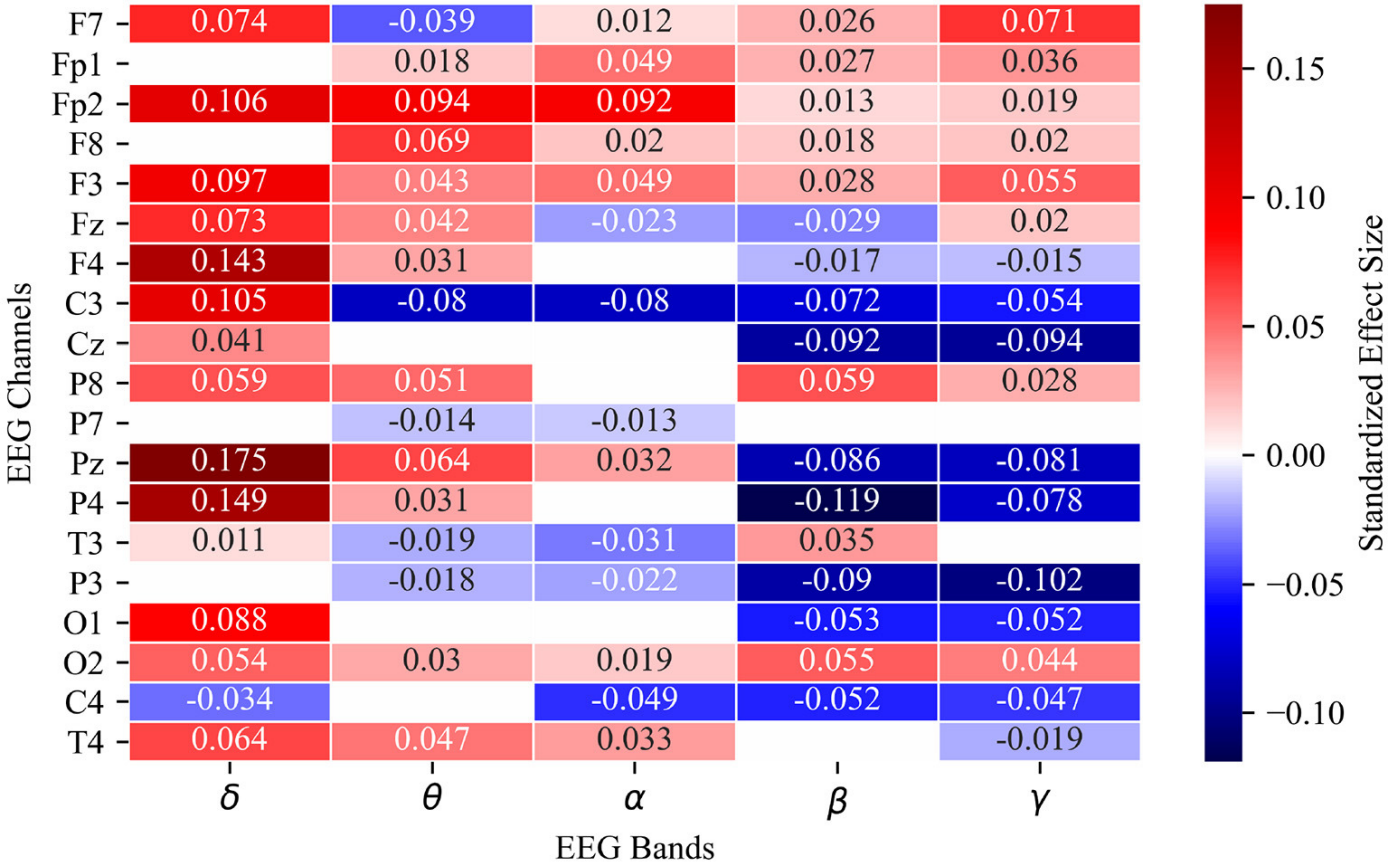
Heat map of estimated fixed effects for 95 linear mixed effects models representing all channel-band combinations of 19 EEG channels and 5 spectral power bands. Fixed effect estimate labeled numerically and by color intensity in each cell. Only significant channel-band combinations are included in heat map. Blank cells did not meet corrected significance threshold of *P* = 0.00009.

Seventy-nine of 95 channel-band power combinations differ significantly between error and non-error. The five largest magnitude standardized effect sizes correspond to δ band power relative to baseline at channels Fp2 (estimate: 0.106, standard error: 0.003, *P* < 2E-16), F4 (estimate: 0.143, standard error: 0.003, *P* < 2E-16), Pz (estimate: 0.175, standard error: 0.004, *P* < 2E-16), and P4 (estimate: 0.149, standard error: 0.004, *P* < 2E-16), and β band power relative to baseline at channel P4 (estimate: –0.119, standard error: 0.003, *P* < 2E-16). Channel-band combinations that show no significant difference between error and non-error are: δ at Fp1, F8, P3, P7; θ at Cz, O1, C4; α at F4, Cz, P8, P4, O1; β at P7, T4; and γ at P7, T3 (Figure 4).

### 3.3. All features analysis

**Figure 5:**
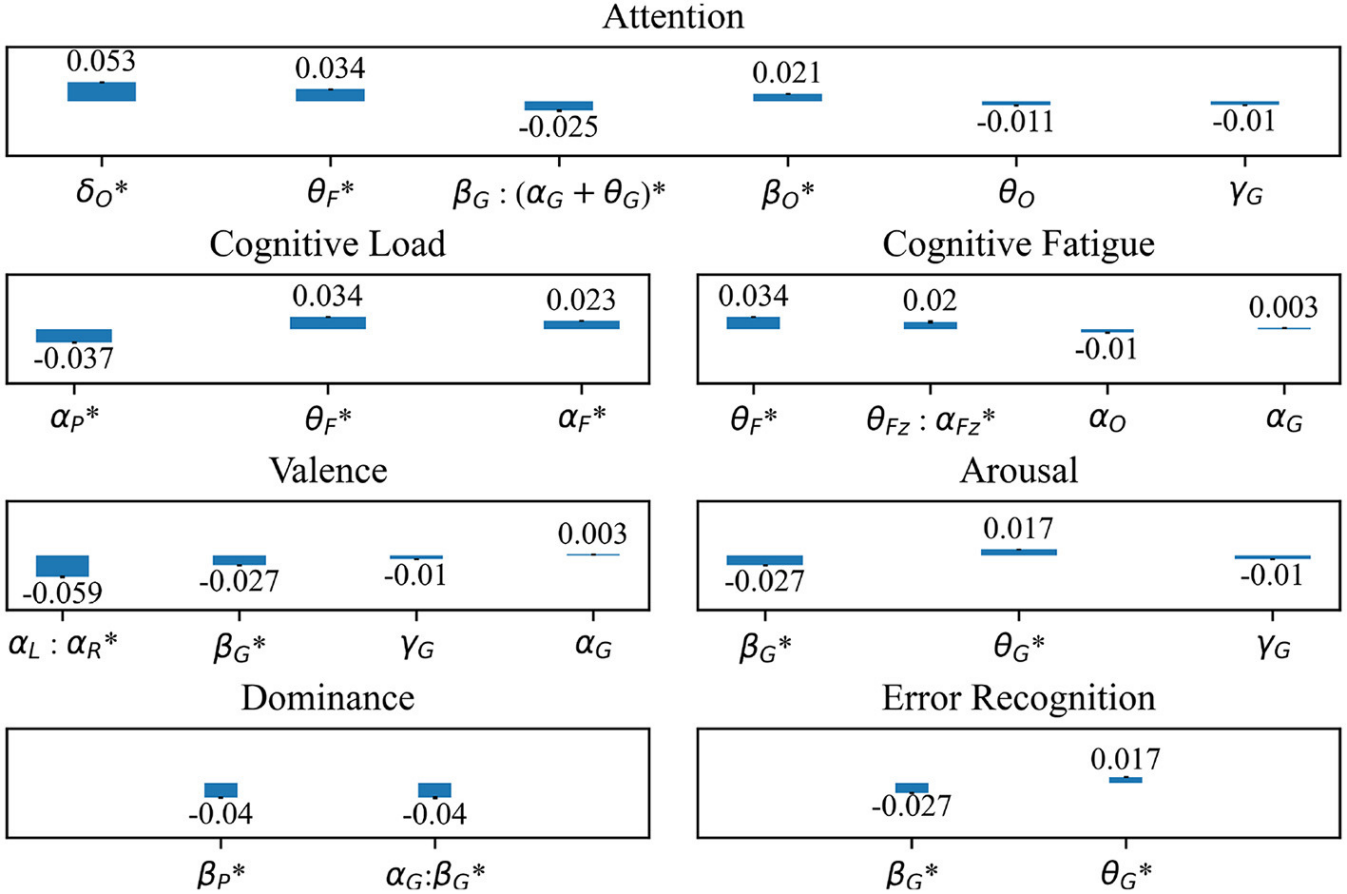
Standardized fixed effect estimates of intraoperative error on previously studied EEG correlates of cognitive and affective features including Attention, Cognitive Load, Cognitive Fatigue, Valence, Arousal, Dominance, and Error Recognition. Electrode regions include O, occipital; F, frontal; G, global; P, parietal; L, left hemisphere; R, right hemisphere. *: Fixed effect estimate meets significance threshold of *P* = 0.00009. All fixed effect estimates are standardized.

Twelve of 15 EEG features differ significantly between error and non-error. For attention, δ power at the occipital electrodes shows the largest magnitude difference between error and non-error (estimate: 0.05, standard error: 0.004, *P* < 2E-16). For cognitive load, α power at the parietal electrodes has the largest magnitude difference (estimate: –0.037, standard error: 0.003, *P* < 2E-16). The most informative feature for cognitive fatigue is θ power at the frontal electrodes (estimate: 0.034, standard error: 0.003, *P* < 2E-16). For valence, the most informative feature is α power asymmetry between the left and right electrodes (estimate: –0.059, standard error: 0.004, *P* < 2E-16). For arousal, β power globally has the largest magnitude difference (estimate: –0.027, standard error: 0.003, *P* < 2E-16). For dominance, global α:β power ratio (estimate: –0.040, standard error: 0.004, *P* < 2E-16) and β power at the parietal electrodes (estimate: –0.040, standard error: 0.003, *P* < 2E-16) have similar effect sizes. The most informative feature for error recognition is global β power (estimate: –0.027, standard error: 0.003, *P* < 2E-16) (Figure 5).

To validate our EEG feature results against potential signals due primarily to EEG pre-processing methods, we compare our full model (a fixed effect of intraoperative error and a random effect of individual variance) with a model that only incorporates the random effect of individual variance in post-processed EEG data. Using a simulation based approach, the likelihood ratio lower bounds for the 95% statistical power C.I.s to detect intraoperative error effects in the full EEG feature models as compared to the simple random effect models ranged from 98.84 to 99.63%. To validate our EEG feature results against potential signals in EEG noise, we compare our full model using post-processed EEG data with our full model using raw EEG data. The likelihood ratio lower bounds for the 95% statistical power C.I.s to detect intraoperative error effects using post-processed EEG data as compared to raw EEG data ranged from 99.3 to 99.6%.

## 4. Discussion

Potential cognitive and affective determinants of surgical performance, and their EEG correlates, have been explored in prior research (Gruzelier, 2014; Guru et al., 2015a; Ndaro and Wang, 2018). This work, however, does not couple EEG analysis with automated surgical error detection to capture high temporal-resolution indicators of intraoperative error. By integrating intraoperative error detection with synchronized EEG analysis, we aimed to characterize the neurophysiology of surgeons during robot-assisted surgery (RAS), particularly during intraoperative error.

To achieve this, we built and deployed a novel RAS data capture and analysis platform to examine the relationships among EEG spectral band powers at various channels, previously proposed EEG correlates of various cognitive and affective features in other contexts, and intraoperative error. Our primary statistical tools, linear mixed effects models (LMMs), are widely used in psychophysiological research particularly because of the non-independence of the data in hierarchical structures (Bagiella et al., 2000; Koerner and Zhang, 2017; Frömer et al., 2018). While sequential 30ms EEG samples may be correlated, we are not examining a temporal effect in our model, so the impact of potential residual error autocorrelation on the error effect is not meaningful for our hypotheses. For this reason, and to our knowledge, the standard LMMs in neurophysiological research do not use autoregressive residual covariance matrices (West et al., 2006). Additionally, when we randomly shuffle our datasets to remove any potential residual autocorrelation that may impact our results, our effect sizes and significance tests remain unchanged.

Our results suggest that relative band powers at most channels demonstrate significant changes during errors as compared to when errors are not being committed. We observe that EEG features which are proposed correlates of attention, cognitive load, cognitive fatigue, valence, arousal, dominance, and error recognition in other contexts (Klimesch, 1999; Strijkstra et al., 2003; Harmony, 2013; Liu and Sourina, 2013; Verma and Tiwary, 2017; Tempel et al., 2020) demonstrate significant changes during intraoperative error and may underlie the error-related signals detected in this study.

The direction of change in EEG features largely aligns with proposed associations between EEG statistics and cognitive or affective features in other contexts. Increases in occipital δ power have been associated with increases in attention (Harmony, 2013), and in our study, these increases are also observed during intraoperative error. Decreases in parietal α power have been associated with decreases in cognitive load (Klimesch, 1999). In our study, decreases in parietal α power were also observed during intraoperative error. Increases in frontal θ power have been associated with increases in cognitive fatigue (Strijkstra et al., 2003), and we observed an increase in frontal θ power during error. Left and right hemisphere α power asymmetry has been proposed as an indicator of emotional valence (Strijkstra et al., 2003) with higher asymmetry corresponding to more positive valence. We observed a decrease in α power asymmetry during error. Decreases in global β power have been associated with increased arousal (Liu and Sourina, 2013), and we observed a decrease in global β power during error. Increases in parietal β power have been associated with increases in dominance (Verma and Tiwary, 2017), and we observed decreases in parietal β power during error. For error recognition, increases in global β power have been associated with increased response inhibition during voluntary motor tasks (Tempel et al., 2020). In our study, global β power decreased during intraoperative error.

While this study attempts to characterize the physiological correlates of intraoperative error, this study is not designed to test the hypothesis that the cognitive and affective states correlate with physiological metrics that are causative of error. The EEG activations described in these results are exploratory and intended to generate hypotheses for future studies that can formally test hypotheses generated from the current findings. Moreover, correlations between physiological signals are not indicative of causation and therefore future studies may attempt to implement causal interventions to test the directional relationship between errors and physiological states.

There are several limitations of this study. First, the number of participants was neither large nor evenly distributed across skill level, potentially skewing cohort analysis. Second, using bandpass filters and RMS power calculations for frame-by-frame channel-band and EEG feature power measurements are accompanied by a tradeoff in the settling time of the filter. Third, the error detection video pipeline we use is based on color and text-based error indicators that appear in the operating console during surgical simulations. These error indicators are part of the built-in simulation software and cannot be turned off or altered during simulation tasks. This presents the possibility that the neurophysiological changes that accompany error may be confounded by a response to the specific error indicator in the stimulator. That said, we observed significant changes in parietal channels that would seem to be less likely if the neurophysiological changes were due to vision-based stimuli alone. Finally, these results may be affected by other variables such as movement at the console and time at the console. Based on our observations, low performers tended to move and shift body positions more frequently than high performers, potentially indicative of discomfort at the console. Low performers also required more time to complete the simulation tasks.

Despite these limitations, this study shows that a novel RAS data capture and analysis platform can enable the detection of distinct neurophysiological changes associated with intraoperative error. These changes are largely consistent with known relationships between EEG metrics and cognitive or affective factors that impact performance. This data highlights the possibility of using objective biometric data capture and error detection to better understand the neurophysiology of intraoperative error and help guide more accurate human performance assessments and precision training for surgeons and other teleoperators.

## Data availability statement

The datasets presented in this article are not readily available because HIPAA. Requests to access the datasets should be directed to CD'A.

## Ethics statement

The studies involving human participants were reviewed and approved by University of California, San Diego School of Medicine Institutional Review Board IRB#: 800026. The patients/participants provided their written informed consent to participate in this study.

## Author contributions

CD'A: conceptualization, software, formal analysis, and writing—original draft. EA-S: supervision and funding acquisition. EH: investigation and project administration. NG: investigation. HC and LA: writing—review and editing, supervision, and funding acquisition. RB: writing—review and editing, project administration, supervision, and funding acquisition. All authors contributed to the article and approved the submitted version.
